# Automated Triage for New Keratoconus Referrals Using Multimodal Deep Learning

**DOI:** 10.1016/j.xops.2026.101208

**Published:** 2026-04-30

**Authors:** Shafi Balal, Lynn Kandakji, Marcello Leucci, Pearse A. Keane, Daniel Gore, Nikolas Pontikos, Bruce D. Allan

**Affiliations:** 1 Moorfields Eye Hospital NHS Trust, London, UK; 2 Institute of Ophthalmology, University College London, London, UK; 3 NIHR Biomedical Research Centre at Moorfields Eye Hospital NHS Foundation Trust, London, UK

**Keywords:** Artificial intelligence, Automation, Keratoconus, Triage

## Abstract

**Purpose:**

To develop and validate deep learning models for predicting keratoconus progression risk using multimodal imaging and clinical data, enabling risk-stratified patient monitoring.

**Design:**

A retrospective cohort study with external validation.

**Participants:**

We analyzed 7396 eyes of 3893 patients (internal dataset) and 963 eyes of 519 patients (external validation dataset) with keratoconus who underwent MS-39 (CSO Italia) anterior-segment OCT (AS-OCT) and Placido topography between October 2020 and June 2024.

**Methods:**

Progression was defined using the global consensus criteria requiring changes in multiple parameters above device-specific precision limits. We compared conventional machine learning, unimodal deep learning, and multimodal fusion architectures to predict 2-year progression risk from baseline data. We developed recurrent neural networks to incorporate data from sequential clinic visits. We assessed clinical utility through simulated risk-stratified triage pathways in an external validation cohort.

**Main Outcome Measures:**

The area under the receiver operating characteristic curve (AUROC), sensitivity, specificity, and predictive values for keratoconus progression within 2 years were measured.

**Results:**

The multimodal model of AS-OCT, Placido, and tabular data yielded the best single-visit prediction (AUROC 0.84, 95% confidence interval [CI]: 0.83–0.85); adding sequential visits via long short-term memory boosted this to 0.93 (95% CI: 0.91–0.96). Fifty-eight percent of patients could be classified as low risk (90% chance of correctly predicting no progression in 2 years) after their baseline clinic visit, rising to 83% with inclusion of data from a second clinic visit.

**Conclusions:**

Artificial intelligence models can be used to develop risk-stratified pathways for monitoring keratoconus, reducing unnecessary follow-up for patients at low risk of progression while ensuring optimal resource allocation and timely intervention for those at high risk.

**Financial Disclosure(s):**

Proprietary or commercial disclosure may be found in the Footnotes and Disclosures at the end of this article.

Keratoconus is a common primary corneal ectasia characterized by progressive thinning and steepening of the cornea, resulting in irregular astigmatism, increased higher-order aberrations, and visual deterioration. While keratoconus typically progresses through childhood and early adulthood before stabilizing by the third or fourth decade, approximately 10% of untreated cases eventually require corneal transplantation.^1^ Corneal cross-linking has emerged as the standard of care for progressive disease, successfully arresting progression in 88%–100% of patients.^2^, ^3^, ^4^, ^5^

Not all newly diagnosed keratoconus patients have progressive disease. In studies of the natural history of keratoconus, rates of progression have been estimated at between 20% and 30%.^6^^,^^7^ Corneal cross-linking also has associated costs and risks: 6% of patients in a US multicenter trial lost 2 lines of corrected distance visual acuity after treatment. Recognized complications include corneal scarring and haze.^8^ Continued monitoring to confirm progression and the need for corneal cross-linking has therefore become standard of care in the contemporary management of keratoconus.^9^^,^^10^

Efficient and safe allocation of available health care resources for disease monitoring requires accurate risk classification at the first visit to help modulate the frequency of subsequent review. In keratoconus, frequent review for low-risk cases can block access to clinical review for cases at high risk of progression, leading to delayed intervention and avoidable loss of vision.^7^^,^^11^ With rising diagnostic rates and finite clinic capacity, the need for reliable triage tools is increasingly urgent at a national level.

Artificial intelligence (AI) models have shown significant promise in the detection of both early and manifest keratoconus.^12^^,^^13^ The ability of AI models to detect patterns in data that are not readily apparent to human observers can also be used to classify disease progression risk.^14^ Deep learning (DL) models, based on the analysis of raw image data, and multimodal models, amalgamating data from more than 1 source, are particularly powerful.^15^^,^^16^

Previous attempts at predicting progression risk in keratoconus using survival analysis or AI modeling have been limited by arbitrary ground-truth definitions of progression, failure to compare alternate models, small datasets, and lack of external validation.^17^, ^18^, ^19^, ^20^, ^21^, ^22^, ^23^, ^24^

We set out to develop and validate DL models to predict 2-year risk of keratoconus progression using baseline data from the initial clinical visit, incorporating both imaging and tabular clinical features from a large multicenter dataset. We then evaluated whether incorporating data from 2 sequential visits (initial review and 1 follow-up visit) using recurrent neural networks could further improve predictive accuracy. Finally, we sought to demonstrate clinical utility by simulating clinical setting triage into risk-stratified monitoring pathways and validated our approach using an external dataset to assess generalizability.

## Methods

### Dataset and Participants

We created internal and external datasets using a retrospectively retrieved data extract from consecutive patients who had MS-39 (CSO Italia) anterior-segment OCT (AS-OCT) scans in a dedicated keratoconus monitoring clinic (The Early Keratoconus Clinic) at Moorfields Eye Hospital, London, UK, between October 2020 and June 2024 (internal dataset) and a similar keratoconus monitoring clinic at Croydon University Hospital National Health Service Trust, Thornton Heath, United Kingdom (external dataset). Because this was an observational study using anonymized data collected in the course of routine clinical practice, individual patient consent was not required.^25^ We obtained ethical approval from the United Kingdom Health Research Authority (ref: 22/PR/0249).^26^ We accessed the datasets through the INSIGHT^27^ virtual research environment (Health Research Authority ref: 20/WS/0087). This study complies with the Declaration of Helsinki.

The datasets comprised 2 imaging modalities: AS-OCT and Placido disk corneal topography images, alongside corresponding tabular features (Table 1). We performed feature engineering to create additional spatial metrics, calculating Euclidean distance from the central corneal axis for thinnest point of stroma and thinnest point of epithelium.

**Table 1:** Baseline Characteristics of Study Cohort

	Internal Dataset (n = 3893)		External Dataset (n = 519)	
	Stable	Progression	Stable	Progression
Number of patients	2920 (75%)	973 (25%)	424 (81.6%)	95 (18.3%)
Male	3284 (59.2%)	1113 (60.2%)	527 (67.0%)	103 (58.4%)
Age	30.89 (26.35–36.22)	27.42 (22.65–33.22)	32.23 (28.35–37.76)	26.67 (21.21–32.18)
Mean follow-up (months)	27.8 ± 4.2	28.5 ± 5.1	24.2 ± 3.8	23.2 ± 2.8
Anterior chamber depth (mm)	3.77 (3.54–4.01)	3.79 (3.58–3.99)	3.75 (3.54–3.96)	3.83 (3.56–4.04)
Central corneal thickness (μm)	488.31 (457.05–519.06)	479.25 (448.08–506.53)	485.11 (447.89–517.47)	472.78 (442.18–508.93)
K_max_ (D)	51.2 (47.8–56.9)	53.1 (49.7–56.9)	51.80 (48.16–57.63)	53.29 (49.41–58.82)
Front K^1^ (D)	44.3 (42.7–46.3)	44.8 (43.1–46.4)	44.52 (42.90–46.55)	44.75 (42.91–46.85)
Front K^2^ (D)	46.9 (45.0–50.1)	47.6 (45.6–50.4)	47.40 (45.31–50.54)	48.18 (45.35–51.21)
Back K^2^ (D)	60.7 (56.1–68.4)	63.3 (58.5–70.0)	5.44 (4.89–5.94)	5.32 (4.84–5.82)
Front coma (D)	1.95 (1.04–3.03)	2.48 (1.51–3.58)	2.14 (1.19–3.57)	2.68 (1.39–3.71)
Front trefoil (D)	0.83 (0.49–1.31)	0.95 (0.61–1.39)	0.88 (0.60–1.34)	1.09 (0.57–1.51)
Front tilt (D)	0.50 (0.25–0.92)	0.70 (0.40–1.15)	0.57 (0.30–1.02)	0.69 (0.37–1.15)
Front spherical aberration (D)	−0.18 (−0.43–0.20)	−0.10 (−0.42–0.31)	−0.18 (−0.38–0.27)	−0.18 (−0.34–0.35)
Front corneal power (D)	51.93 (54.49–50.13)	52.26 (54.34–50.45)	52.02 (54.78–50.14)	52.34 (55.14–50.26)
Front HOA RMS (D)	2.47 (1.46–3.86)	3.05 (2.03–4.37)	2.76 (1.67–4.25)	3.31 (1.85–4.52)
White	596 (19.3%)	202 (20.8%)	60 (14.0%)	20 (21.1%)
Asian	403 (13.1%)	162 (16.7%)	76 (17.7%)	9 (9.5%)
Black	204 (6.6%)	87 (9.0%)	50 (11.7%)	12 (12.6%)
Other	374 (12.1%)	113 (11.6%)	31 (7.2%)	4 (4.2%)
Unknown	1343 (43.5%)	409 (42.1%)	207 (48.3%)	50 (52.6%)

D = diopter (equivalent dioptric power derived from corneal wavefront error over a 6 mm diameter, using polar expression of Zernicke terms); HOA RMS = higher order aberrometry root mean square; K^1^ = flattest meridian; K^2^ = steepest meridian; K_max_ = maximum anterior keratometry.

Both eyes of all patients over the age of 18 with a diagnosis of keratoconus were eligible for inclusion if they had at least 2 scans 90 days apart with a minimum of 2 years follow-up. We excluded eyes with prior cross-linking, refractive surgery, or keratoplasty. We based keratoconus diagnosis on clinical examination and corneal tomography. While the MS-39 disease classification algorithm^28^ was utilized to indicate the probability of keratoconus, algorithm results alone were not considered diagnostic. In each case, the diagnosis was confirmed by an experienced clinician. Follow-up intervals were determined by the treating clinician (range 3–12 months) based on recognized risk factors for keratoconus progression—younger age, non-White European ethnicity, and advanced disease in the contralateral eye.^20^

### Instrument and Procedure

We obtained all data using the MS-39 corneal tomographer (CSO Italia), which employs a combination of spectral-domain OCT tomography and Placido-disk topography. In the default acquisition mode, this included a single Placido top-view image and a set of 12 or 25 radial spectral-domain OCT scans.^29^ Both the 12 × 10 mm and 25 × 16 mm modalities were included as we found no differences in intertest measurement variation for a range of corneal tomographic parameters between these alternate scan modalities.^30^ From the 12 or 25 radial slices, we selected the slice containing the thinnest corneal point. The manufacturer’s instructions were followed by all operators, and all measurements were made in mesopic luminance. We discarded scans flagged as poor quality by the manufacturer’s acquisition software at the time of data acquisition or filtered poor-quality scans from the anonymized dataset using the same criteria: Placido coverage <65% or section coverage <85%.

### Ground Truth Labeling

Recent clinical trials have defined disease progression in keratoconus using static tomographic threshold values.^2^^,^^4^^,^^6^^,^^8^^,^^31^, ^32^, ^33^, ^34^, ^35^, ^36^, ^37^, ^38^, ^39^, ^40^, ^41^, ^42^ These thresholds are often notional: for example, a 1-diopter increase in steep anterior keratometry. We based the ground truth label on the 2015 global consensus statement on keratoconus, which defines progression as change in at least 2 of 3 parameter categories (anterior curvature, posterior curvature, or thinning), where the magnitude of change in the parameters measured is above the precision limits of the testing system used.^43^ Our ground truth definition for keratoconus progression is based on a comprehensive analysis of MS-39 measurement precision—tables of the thresholds for each metric can be found in our previous work.^30^ We applied the 2015 consensus statement definition using the mean of 3 consecutive measures to derive referent values for each parameter at each timepoint, and adaptive threshold values for keratoconus progression, which account for deterioration in MS-39 corneal tomographic measurement precision with advancing disease severity.^30^ For example, the threshold for maximum anterior keratometry ranged from 0.8 diopter in mild disease to 3D in advanced disease, reflecting the nonlinear decline in measurement precision with increasing corneal irregularity.

### Preprocessing

The preprocessing pipeline included normalization of pixel intensity values in the images to the range [0, 1], resizing to 224 × 224 pixels and using bilinear interpolation. We preserved the aspect ratio during resizing and normalized each image individually based on its minimum and maximum pixel values to enhance local contrast and structural details. Data augmentation included random rotation (±30°) and horizontal flipping. We implemented a patient-based split strategy to ensure all images from the same patient were assigned to the training, validation, or test set, preventing data contamination. As each patient had 3 repeated scans (2 at 25 × 16 and 1 at 10 × 12) for each eye on the same visit date, we selected a single random image for each eye for model training. Isolation of the cornea from AS-OCTs was evaluated using Google’s Segment Anything Model but did not improve model performance and was not included in the final analysis in order to reduce the computational burden.^44^

### Data Partition

We divided patients in the internal dataset into training (85%) and test (15%) datasets using stratified sampling to maintain a uniform age distribution. We trained and validated DL models using fivefold cross-validation within the (85%) training dataset, ensuring all data from the same patient remained within the same fold. The separate (15%) test dataset was then used to derive performance metrics for model evaluation.

### Prediction of 2-Year Progression Risk from Baseline Data

We compared a range of modeling techniques aiming to predict keratoconus progression within 2 years from data collected at the first clinical visit: (1) conventional machine learning using tabular data; (2) unimodal DL using raw Placido or corneal-sectional OCT images; and (3) multimodal fusion architectures combining imaging and tabular data. The best-in-class model for each category was selected using the area under the receiver operator characteristic curve (AUROC).

#### Machine Learning Models on Tabular Data

The baseline comparator was a logistic regression model. We tested several other machine learning models: support vector machines, decision trees, random forest, and gradient boosting (XGBoost). We considered all available processed numeric corneal tomographic outputs from the MS-39 as candidate independent variables for the machine learning models and performed feature selection using ablation studies. The final variables included in the machine learning models are summarized in Table 1. We standardized selected features and addressed class imbalance using synthetic minority oversampling.^45^

#### Deep Learning Models on Placido and Cross-Sectional OCT Images

We evaluated unimodal DL models using either Placido images or individual AS-OCT sections through the thinnest corneal point. We passed images through several different convolutional neural network (CNN) architectures: (1) a custom CNN with four convolutional layers with batch normalization followed by a fully connected layer; (2) a deeper CNN comprising 12 convolutional layers organized in five architectural blocks for hierarchical feature extraction (all using 3 × 3 kernels with padding), and (3) transfer learning with ImageNet-pretrained ResNet50,^46^ EfficientNet-B7,^2^ and Vision Transformer (ViT-B/16).^5^ We followed a 2-phase approach for transfer learning: initially freezing the backbone while training the classification head for 5 epochs (i.e., 5 complete passes of the entire training dataset through the algorithm), then unfreezing all layers for fine-tuning with a reduced learning rate.

#### Multimodal Deep Learning Models

We examined 3 multimodal approaches to integrate OCT, Placido, and tabular data: early fusion, concatenating raw image features and tabular data before classification; intermediate fusion, combining modality-specific learned representations via multihead attention; and late fusion, using weighted ensemble averaging of separate per-modality classification outputs.

#### Model Training Parameters

We trained models for 50 epochs with early stopping implemented at a patience of five epochs (i.e., 5 additional epochs were allowed after the validation performance had stopped improving before training was halted) using Adam optimization with an initial learning rate of 0.0001 and a batch size of 64. We used an output probability threshold of 0.5 for binary classification (progression or no progression) across all models. We addressed class imbalance using weighted binary cross-entropy loss for DL models, with positive class weights calculated as the ratio of negative to positive samples. We performed hyperparameter optimization using a random grid search. We evaluated all models using group k-fold cross-validation (k = 5) and developed using PyTorch (version 1.12.1). We performed all model training in the INSIGHT virtual research environment using NVIDIA T4 GPUs (16GBx2).

### Prediction of 2-Year Progression Risk from Sequential Visit Data

We then evaluated whether predictive performance could be improved beyond the first-visit baseline data models by incorporating tabular data from a second clinic visit. We implemented 2 recurrent neural network architectures to capture temporal dependencies for cases with sequential visits, where no progression had occurred at the second visit. We developed both gated recurrent unit and long short-term memory (LSTM) networks with attention mechanisms to prioritize relevant features across visits when predicting progression outcomes. Additionally, we implemented a transformer architecture to help capture long-range dependencies. All models were trained to predict final progression status from data captured at the first 2 clinical review visits. Training consisted of 50 epochs with early stopping based on validation performance, using batch sizes of 32 and Adam optimization with an initial learning rate of 0.001.

### Simulated Clinical Setting Triage

Artificial intelligence classification models return an output probability between 0 and 1. For translation into clinical use, output probability thresholds need to be set to define classification category boundaries with reference to predictive value.

Predictive value—the proportion of cases assigned to a risk category who truly have the outcome—varies with the classification threshold applied to model output probability. Unlike sensitivity and specificity, predictive value is intuitive for patients, directly conveying the percentage chance of progression within their assigned risk category.

We simulated triage into 3 risk categories designed to help modulate the clinic review schedule for patients with a new diagnosis of keratoconus: low, moderate, and high risk. Output probability thresholds for each of these categories were calculated using the best-performing baseline AI algorithm. Low risk was defined as an output probability returning a negative predictive value (NPV) ≥90% (≥90% probability of correctly predicting no keratoconus progression within 2 years). High risk was defined as an output probability returning a positive predictive value ≥90% (≥90% probability of correctly predicting keratoconus progression within 2 years). At the second clinic visit, we refined our baseline classification for patients with no progression who were initially in the moderate-risk category (NPV ≤ 90%; positive predictive value ≤ 90% at the baseline visit) by applying our best-performing sequential visit data model. To help estimate the impact of applying the best-performing models to clinical setting triage, the numbers of patients allocated to each risk category after the first and second clinic visits were then analyzed using the external validation dataset.

### Model Explainability

We applied model-specific techniques to interpret predictors of progression. For XGBoost models, we utilized Shapley Additive Explanations feature importance ranking.^47^ The CNN-based models were interpreted using gradient-weighted class activation mapping^48^ to generate saliency maps.

### Statistical Analysis

Both the AUROC and area under the precision–recall curve were computed to take account of class imbalance. To facilitate comparison with existing models of keratoconus progression risk, sensitivity and specificity were calculated at an output probability of 0.5 for each of the new models developed here. Confidence intervals (CIs) were obtained via resampling with 2000 bootstraps.

## Results

### Patient Characteristics

In total, 7396 eyes of 3893 patients were present in the internal dataset, of which 1849 (25%) demonstrated keratoconus progression in the available follow-up period. For the external dataset, 963 eyes of 519 patients were available, of which 176 eyes (18.3%) progressed. The mean visit to first detection of progression was 4.2 ± 2.1 months for the internal dataset and 3.9 ± 2.8 months for the external dataset. Other baseline characteristics for both internal and external datasets are summarized in Table 1.

### Prediction of 2-Year Progression Risk from Baseline Data

**Figure 1:**
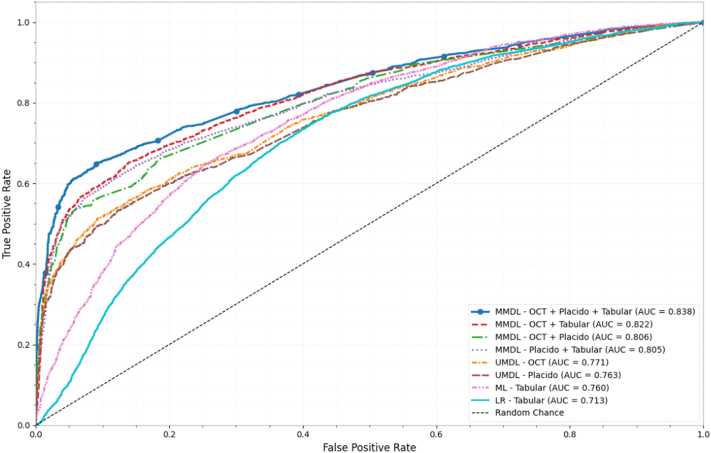
Receiver operating characteristic curves comparing performance of different model architectures and data modality combinations for predicting 2-year progression risk from first-visit baseline data. AUC = area under the curve; LR = logistic regression; ML = machine learning; MMDL = multimodal deep learning; UMDL = unimodal deep learning.

A multimodal intermediate fusion DL architecture combining OCT, Placido, and tabular data was the best-performing model for predicting risk of keratoconus progression within 2 years using data available at the baseline clinic visit (AUROC = 0.84, 95% CI: 0.83–0.85). Performance in this task for comparator models is summarized in Figure 1 and Table 2. The same multimodal DL model also performed best in the external validation dataset (AUROC = 0.75, 95% CI: 0.74–0.76).

**Table 2:** Performance Metrics of First-Visit Baseline Data Predictive Models for 2-Year Progression Risk on Internal and External Test Datasets

	Internal Dataset				External Dataset			
	AUROC	AUPRC	Sensitivity	Specificity	AUROC	AUPRC	Sensitivity	Specificity
OCT and Placido and tabular	0.84 (0.83–0.85)	0.71 (0.69–0.73)	0.76 (0.74–0.78)	0.82 (0.81–0.83)	0.75 (0.74–0.76)	0.49 (0.46–0.51)	0.82 (0.81–0.84)	0.59 (0.57–0.62)
OCT and tabular	0.82 (0.81–0.83)	0.69 (0.67–0.71)	0.78 (0.76–0.80)	0.76 (0.74–0.78)	0.72 (0.72–0.73)	0.52 (0.49–0.55)	0.85 (0.83–0.87)	0.55 (0.51–0.59)
OCT and Placido	0.81 (0.79–0.82)	0.67 (0.65–0.69)	0.82 (0.80–0.84)	0.71 (0.69–0.73)	0.73 (0.73–0.74)	0.51 (0.48–0.54)	0.78 (0.76–0.80)	0.58 (0.57–0.59)
Placido and tabular	0.81 (0.79–0.82)	0.68 (0.66–0.70)	0.75 (0.73–0.77)	0.78 (0.76–0.80)	0.71 (0.70–0.72)	0.54 (0.52–0.56)	0.66 (0.65–0.68)	0.66 (0.66–0.67)
CNN OCT	0.77 (0.76–0.78)	0.63 (0.61–0.65)	0.74 (0.72–0.76)	0.72 (0.70–0.74)	0.69 (0.68–0.69)	0.47 (0.44–0.49)	0.78 (0.76–0.80)	0.52 (0.51–0.53)
CNN Placido	0.76 (0.75–0.78)	0.62 (0.60–0.64)	0.79 (0.77–0.81)	0.66 (0.64–0.68)	0.69 (0.68–0.70)	0.47 (0.44–0.50)	0.74 (0.72–0.76)	0.56 (0.55–0.57)
Tabular (XGBoost)	0.76 (0.75–0.77)	0.61 (0.59–0.63)	0.81 (0.79–0.83)	0.63 (0.61–0.65)	0.68 (0.67–0.69)	0.46 (0.43–0.49)	0.81 (0.79–0.83)	0.47 (0.46–0.48)
Tabular (logistic regression)	0.71 (0.70–0.72)	0.54 (0.52–0.56)	0.69 (0.67–0.71)	0.69 (0.67–0.71)	0.63 (0.62–0.64)	0.42 (0.39–0.45)	0.72 (0.70–0.74)	0.58 (0.57–0.59)

Values represent mean with 95% confidence intervals in parentheses. Parentheses contain 95% confidence intervals.

AUPRC = area under the precision–recall curve; AUROC = area under the receiver operating characteristic curve; CNN = convolutional neural network.

### Prediction of 2-Year Progression Risk from 2 Sequential Visits

**Figure 2:**
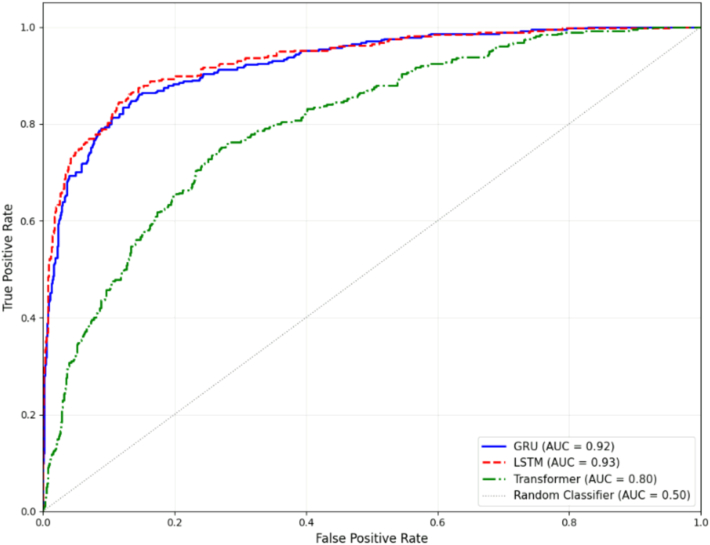
Receiver operating characteristic curves comparing performance of different model architectures for predicting 2-year progression risk from sequential visit data. AUC = area under the curve; GRU = gated recurrent unit; LSTM = long short-term memory.

When utilizing combined (sequential visits) data from both the first and second visits, the LSTM model achieved the highest performance across all metrics, with AUROC of 0.93 and area under the precision–recall curve of 0.88, while maintaining a sensitivity of 0.73 and specificity of 0.96 (Table 3). The AUROCs for each model are displayed in Figure 2.

**Table 3:** Performance Comparison of Recurrent Neural Network and Transformer Architectures for Disease Progression Prediction Using Sequential Visit Data

	Internal Dataset				External Dataset			
	AUROC	AUPRC	Sensitivity	Specificity	AUROC	AUPRC	Sensitivity	Specificity
LSTM	0.93 (0.91–0.96)	0.88 (0.86–0.90)	0.73 (0.68–0.77)	0.96 (0.95–0.97)	0.90 (0.85–0.95)	0.82 (0.78–0.86)	0.86 (0.81–0.91)	0.83 (0.80–0.87)
GRU	0.92 (0.91–0.95)	0.87 (0.85–0.89)	0.69 (0.67–0.72)	0.95 (0.93–0.96)	0.86 (0.80–0.90)	0.77 (0.74–0.80)	0.82 (0.78–0.86)	0.80 (0.74–0.86)
Transformer	0.84 (0.83–0.85)	0.73 (0.71–0.74)	0.64 (0.62–0.66)	0.87 (0.85–0.89)	0.78 (0.72–0.85)	0.65 (0.60–0.71)	0.74 (0.70–0.78)	0.72 (0.65–0.79)

Values are presented as mean with 95% confidence intervals in parentheses.

AUROC = area under the receiver operating characteristic curve; AUPRC = area under the precision–recall curve; GRU = gated recurrent unit; LSTM = long short-term memory.

### Simulated Clinical Setting Triage

**Figure 3:**
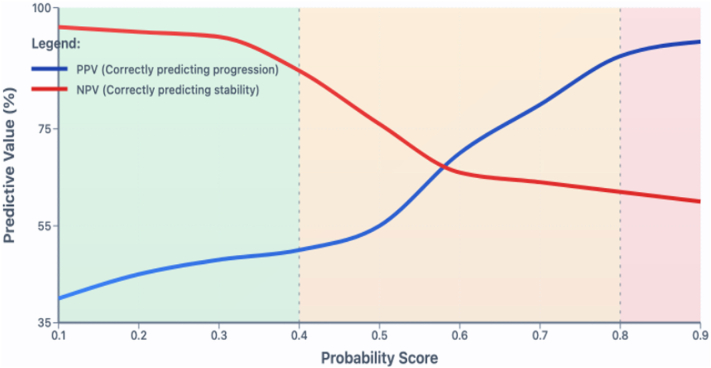
Predictive value versus output probability score for a multimodal deep learning algorithm predicting the risk of keratoconus progression from data gathered at the baseline clinic visit. Low risk (green) = <0.4, moderate risk (yellow) = 0.4-0.8, and high risk (red) = >0.8. NPV = negative predictive value; PPV = positive predictive value.

**Figure 4:**
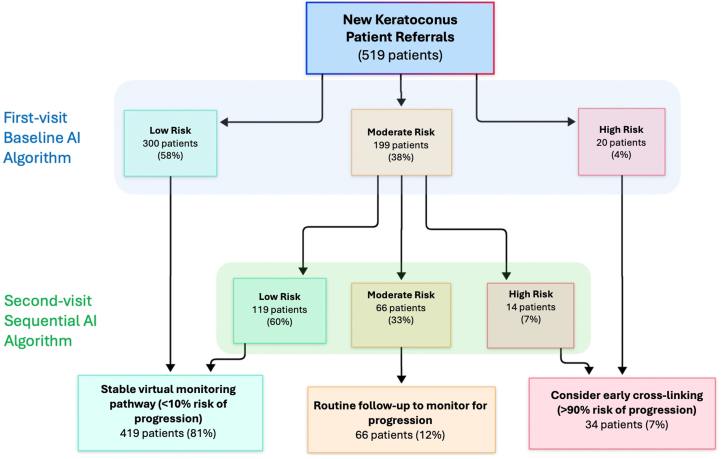
Risk stratification and management pathway for keratoconus patients using AI algorithms. The flowchart illustrates the clinical decision-making process for the 519 keratoconus patients in the external validation dataset, showing how baseline and sequential visit AI algorithms classify patients into risk categories and guide subsequent management strategies. AI = artificial intelligence.

The best baseline and sequential visit algorithms were combined to simulate clinical setting triage on the external validation dataset. For the baseline AI algorithm, the positive predictive value was > 90% (high-risk patients; 95% CI: 88%–92%) at an output probability >0.8 and an NPV was > 90% (low-risk patients; 95% CI: 89%–91%) at an output probability of < 0.4 (Fig 3). From the first visit, 300 patients (58%) could be triaged as low risk. Using sequential data from the first and second clinic visits, this was increased to 437 patients (83%) (Fig 4).

### Model Explainability

**Figure 5:**
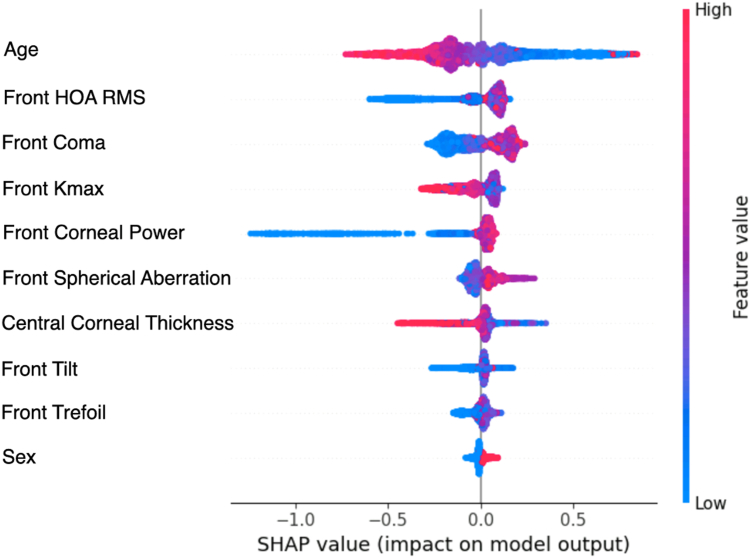
Shapley additive explanations (SHAP) feature importance for predicting progression using the best performing machine learning model (XGBoost) trained on tabular data from the first clinic visit. Features are ranked by their average absolute SHAP value, with the most influential parameters at the top. The density (thickness) of each distribution shows the concentration of data points, while colors represent feature values: red indicates high values, blue indicates low values. For example, older age (red dots) clusters toward negative SHAP values, indicating that higher age reduces the likelihood of progression. K_max_ = maximum anterior keratometry; HOA RMS = higher order aberrometry root mean square; SHAP = Shapley additive explanations.

**Figure 6:**
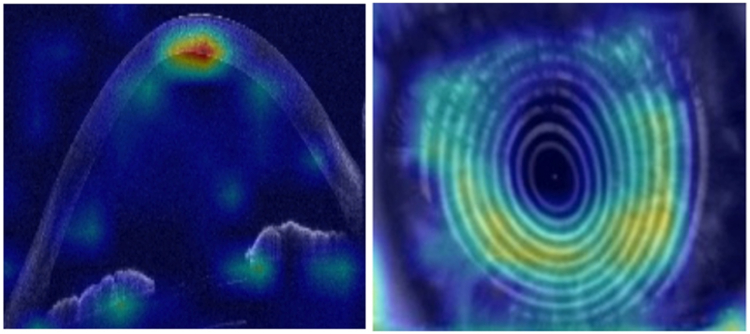
Visualization of the important regions in the convolutional neural network model predictions for progression from first-visit baseline data for OCT and Placido images. The posterior corneal region was consistently highlighted as important for prediction on OCT sections, whereas topography images showed predictions were derived from inferior Placido reflections—consistent with the typical location of cone development.

Shapley Additive Explanations feature importance scores from using first-visit baseline tabular data in the XGBoost model showed age as the dominant progression predictor (Fig 5). Age (AUROC = 0.63, 95% CI: 0.62–0.64), Kmax (AUROC = 0.55, 95% CI: 0.51–0.59), thinnest corneal thickness (AUROC = 0.53, 95% CI: 0.49–0.57), and gender (AUROC = 0.52, 95% CI: 0.50–0.53) used in isolation as single features in the XGBoost model were inferior to the model containing all features (AUROC = 0.76, CI: 0.75–0.77). For the saliency maps, while both AS-OCT and Placido images highlight the central cornea as important for progression risk, the inferocentral regions, reflecting the commonest location of the cone apex, were highlighted in the Placido maps, whereas the posterior cornea is highlighted in the AS-OCT maps (Fig 6).

## Discussion

Our multimodal DL model demonstrated robust predictive performance for 2-year keratoconus progression using baseline data alone (AUROC = 0.84). The integration of sequential visit data using recurrent neural network architectures substantially improved predictive accuracy to AUROC = 0.93. In simulated clinical setting triage using the external validation dataset, this approach enabled classification of up to 83% of patients as low risk (>90% NPV) by their second visit. Current guidelines recommend frequent monitoring for all keratoconus patients, typically every 3 to 6 months, to detect progression.^49^ Our risk-stratified approach could extend monitoring intervals safely for low-risk patients, while prioritizing resources for high-risk cases requiring closer surveillance. Translation into clinical practice will require clear thinking about the levels of acceptable risk versus efficient use of the available health care resource. At minimum, modeling risk with greater accuracy helps to provide a more informed background for the decisions required.

This study builds on our previous work^30^ to establish a rational ground truth definition for keratoconus progression based on mapping precision limits for MS-39 corneal tomography through the measurement range in keratoconus and combining repeated measures. Thresholds for progression were then based on change, greater than accurately defined measurement noise, in 2 or more parameters—as suggested in the 2015 keratoconus consensus.^43^ In addition to accurate labeling for model training, other strengths of this study in comparison with previous AI modeling to predict keratoconus progression risk include the use of a large multicenter dataset with external validation in a distinct patient sample. We investigated a range of models, from logistic regression to multimodal fusion architectures, and demonstrated that data from a second visit can be used in combination with data from the first visit to further improve triage ability using recurrent neural networks.

The ability of the multimodal fusion model to achieve substantially better performance than age alone (AUROC 0.84 vs. 0.63) indicates that it captures an array of biosignals marking keratoconus progression risk, some of which may not have been recognized previously or captured in models based on commonly used corneal tomographic parameters.

There is a trade-off between model power and explainability. The advantage of machine learning models such as XGBoost based on tabular data is that feature analysis techniques, such as Shapley Additive Explanations analysis (Fig 5), help to identify and rank clinically useful determinants of progression risk. Our findings align well with established risk factors for progression (age, more advanced disease) but with an emphasis on higher order aberrations, notably coma, as markers for progression that have not previously been modeled.^20^ Saliency maps from DL models (Fig 6) consistently highlighted the posterior corneal surface and inferocentral regions as critical for classification. This corroborates emerging evidence that posterior changes may precede detectable anterior surface features as early indicators of progression^18^^,^^50^ and aligns with findings from a previous AI study.^17^ The pattern may reflect the compensatory role of epithelial remodeling in masking early anterior curvature changes in keratoconus.^51^

We included both eyes from eligible patients in the dataset used for model training. To determine whether the inclusion of paired eye data had any detrimental effect on discriminant power, we retrained our two leading models (multimodal DL for single visit data and LSTM for sequential visit data) using data from 1 randomly selected eye per patient. Results for the single eye models were similar to those for the same models trained on paired eye data, but with a weak trend to reduced discriminant power. Mean absolute decreases in AUROC for the models trained on single eye data were 0.01 for the multimodal DL model and 0.02 for the LSTM model.

Our primary training cohort did not include pediatric cases (patients under 18 years of age) as this a separate patient stream in the UK. In further sensitivity analyses, both our leading models maintained good performance in a separate test cohort of 340 pediatric patients (age <18 years, mean 16.2 ± 0.82 years) under active monitoring for keratoconus progression with no prior surgical intervention (AUROC 0.84 for multimodal DL; 0.93 for LSTM).

The external cohort was older (32.5 ± 11.6 vs. 30.7 ± 8.2 years, *P* = 0.018), had a lower progression rate (18.3% vs. 25%, *P* < 0.001), and differed significantly in six corneal parameters (*P* < 0.05). Principal component analysis confirmed a genuine distributional shift across tomographic features, with the first 3 components explaining 74.9% of variance despite substantial overlap between cohorts—underscoring the value of external validation in testing model generalizability. Consistent with these differences, model performance was reduced in the external dataset, with mean absolute decreases in AUROC of 0.09 for multimodal DL (baseline visit data) and 0.03 for LSTM (sequential visit data).

These findings should be interpreted within the emerging understanding that AI models in health care inevitably exhibit at least some site-specific performance variation.^52^ A period of silent running, in which newly deployed AI models run in parallel with standard clinical practice without influencing treatment decisions, can reveal clinical setting performance characteristics that may not be captured adequately in retrospective analyses, including robustness to variations in image quality, operator technique, and patient populations.^52^ Silent prospective validation also allows for model updating and recalibration rather than complete redevelopment.^53^

Wherever possible, recurring local validation should include mapping output probability values against predictive values using a dataset derived from the local patient population (Fig 3). Despite the observed decrease in model performance, we were still able to assign over 80% of patients in the external validation dataset to a low-risk category (≥90% NPV) by their second visit using this methodology (Fig 4). This underlines the potential clinical utility and cost-saving potential for the models we have developed, but further work is required in partnership with the device provider on software integration and tools to assist in local calibration before these new risk stratification tools can be made available for routine clinical use.

Finding the right balance between failing to detect progression in low-risk cases by spacing monitoring visits too widely and minimizing the waste of health care resource by spacing monitoring visits far enough apart is a particular challenge in the deployment of models designed to modulate review frequency based on the classification of disease progression risk. As recommended in the recent FAIR-AI implementation guidelines,^54^ we adjusted model output probability thresholds to achieve 90% predictive value to help estimate the proportion of our patients falling into each risk category after 1 and 2 visits (Fig 4). This threshold and downstream appointment allocation based on risk classification could be adjusted based on local clinical priorities, resource constraints, and the extent of any visual loss in cases where keratoconus progression still occurs between visits.

This study has several limitations. The mean follow-up period of 2 years may not capture all progression patterns, particularly slower-developing cases. However, this timeframe supports actionable decisions, enabling early intervention for high-risk eyes while allowing low-risk eyes to be safely assigned to less intensive monitoring.

Our models cannot account for unpredictable factors such as eye rubbing behavior or hormonal changes that may influence disease progression.^55^ The requirement for AS-OCT and Placido topography may limit implementation in resource-constrained settings, though emerging smartphone-based Placido imaging technologies^56^ offer promise for broader accessibility given our demonstration that modeling based on raw Placido images alone can also predict progression with a good level of accuracy (Table 2). The study was limited to 1 device, but the methods and model architectures, including utilization of sequential visit data in recurrent neural networks, are generally applicable. Future studies could look at translation to other corneal tomography devices, and could also include corneal biomechanical data, which may contain additional features relevant to keratoconus progression risk.^57^

In conclusion, this study demonstrates that our multimodal AI approach can effectively predict keratoconus progression from baseline clinical data, with sequential visit recurrent neural network models achieving even greater accuracy. Accurate risk classification provides a rational basis for the allocation of health care resources, reducing the burden of frequent monitoring in low-risk cases and promoting timely access to treatment in high-risk cases. Growing evidence that AI systems can automate the entire keratoconus care pathway—from initial diagnosis^12^^,^^28^ to risk-based triage and automated threshold-driven virtual monitoring^30^—represents a paradigm shift toward more efficient, digitally enabled eye care.

## References

[1] Gordon M.O., Steger-May K., Szczotka-Flynn L. (2006). Baseline factors predictive of incident penetrating keratoplasty in keratoconus. Am J Ophthalmol.

[2] Wittig-Silva C., Chan E., Islam F.M. (2014). A randomized, controlled trial of corneal collagen cross-linking in progressive keratoconus: three-year results. Ophthalmology.

[3] Caporossi A., Mazzotta C., Baiocchi S., Caporossi T. (2010). Long-term results of riboflavin ultraviolet a corneal collagen cross-linking for keratoconus in Italy: the Siena eye cross study. Am J Ophthalmol.

[4] O'Brart D.P., Chan E., Samaras K. (2011). A randomised, prospective study to investigate the efficacy of riboflavin/ultraviolet A (370 nm) corneal collagen cross-linkage to halt the progression of keratoconus. Br J Ophthalmol.

[5] Gore D.M., Leucci M.T., Koay S.-Y. (2021). Accelerated pulsed high-fluence corneal cross-linking for progressive keratoconus. Am J Ophthalmol.

[6] Larkin D.F., Chowdhury K., Burr J.M. (2021). Effect of corneal cross-linking versus standard care on keratoconus progression in young patients: the KERALINK randomized controlled trial. Ophthalmology.

[7] Koppen C., Jiménez-García M., Kreps E.O. (2024). Definitions for keratoconus progression and their impact on clinical practice. Eye Contact Lens.

[8] Hersh P.S., Stulting R.D., Muller D. (2017). United States multicenter clinical trial of corneal collagen crosslinking for keratoconus treatment. Ophthalmology.

[9] Salmon H., Chalk D., Stein K., Frost N. (2015). Cost effectiveness of collagen crosslinking for progressive keratoconus in the UK NHS. Eye.

[10] Chan E, Snibson GR (2013). Current status of corneal collagen cross-linking for keratoconus: a review. Clin Exp Optometry.

[11] Romano V., Vinciguerra R., Arbabi E.M. (2018). Progression of keratoconus in patients while awaiting corneal cross-linking: a prospective clinical study. J Refract Surg.

[12] Hashemi H., Doroodgar F., Niazi S. (2024). Comparison of different corneal imaging modalities using artificial intelligence for diagnosis of keratoconus: a systematic review and meta-analysis. Graefe's Archive Clin Exp Ophthalmol.

[13] Maile H., Li J.-P.O., Gore D. (2021). Machine learning algorithms to detect subclinical keratoconus: systematic review. JMIR Med Inform.

[14] Nderitu P., Nunez do Rio J.M., Webster L. (2024). Predicting 1, 2 and 3 year emergent referable diabetic retinopathy and maculopathy using deep learning. Commun Med.

[15] Venugopalan J., Tong L., Hassanzadeh H.R., Wang M.D. (2021). Multimodal deep learning models for early detection of Alzheimer's disease stage. Sci Rep.

[16] Kline A., Wang H., Li Y. (2022). Multimodal machine learning in precision health: a scoping review. NPJ Digital Med.

[17] Hartmann L.M., Langhans D.S., Eggarter V. (2024). Keratoconus progression determined at the first visit: a deep learning approach with fusion of imaging and Numerical clinical data. Transl Vis Sci Technol.

[18] Kamiya K., Ayatsuka Y., Kato Y. (2021). Prediction of keratoconus progression using deep learning of anterior segment optical coherence tomography maps. Ann Transl Med.

[19] Kato N., Masumoto H., Tanabe M. (2021). Predicting keratoconus progression and need for corneal crosslinking using deep learning. J Clin Med.

[20] Tuft S.J., Moodaley L.C., Gregory W.M. (1994). Prognostic factors for the progression of keratoconus. Ophthalmology.

[21] Maile H.P., Li J.-P.O., Fortune M.D. (2022). Personalized model to predict keratoconus progression from demographic, topographic, and genetic data. Am J Ophthalmol.

[22] Quartilho A., Gore D.M., Bunce C., Tuft S.J. (2020). Royston– Parmar flexible parametric survival model to predict the probability of keratoconus progression to corneal transplantation. Eye.

[23] Ozalp O., Ozaslan M.K., Apaydin F., Karaatli M., Karaman B.B., Yildirim N., Dogan C., Atalay E. (2025). Evaluating Natural Progression of Keratoconus in Relation to Age, Gender, and Disease Severity at Presentation. Cornea.

[24] Atalay E, Özdemir F, Bilgeç MD, Özalp O (2025). Association between corneal densitometry at initial presentation and future progression across age groups in keratoconus. Ophthalmic Physiol Opt.

[25] U. GDPR Information to be provided where personal data have not been obtained from the data subject. https://gdpr-info.eu/art-14-gdpr/.

[26] Health Research Authority What approvals do I need? [Internet]. Health Research Authority. https://www.hra.nhs.uk/approvals-amendments/what-approvals-do-i-need/.

[27] Bilton E.J., Guggenheim E.J., Baranyi B. (2023). A datasheet for the INSIGHT university hospitals Birmingham retinal vein occlusion data set. Ophthalmol Sci.

[28] Del Barrio J.L.A., Eldanasoury A.M., Arbelaez J. (2024). Artificial neural network for automated keratoconus detection using a combined placido disc and anterior segment optical coherence tomography topographer. Transl Vis Sci Technol.

[29] Venkataraman A.P., Domínguez-Vicent A., Selin P. (2022). Precision of a new SS-OCT biometer to measure anterior segment parameters and agreement with 3 instruments with different measurement principles. J Cataract Refract Surg.

[30] Balal S., Cai Y., Kandakji M.L., Liu S., Mullholand P.J., Leucci M.M., Pontikos N., Gore D., Allan B. (2025). Establishing the ground truth for keratoconus progression: combining repeated measures and adapting precision limits to disease severity in tomography. J Cataract Refract Surg.

[31] Al Fayez M.F., Alfayez S., Alfayez Y. (2015). Transepithelial versus epithelium-off corneal collagen cross-linking for progressive keratoconus: a prospective randomized controlled trial. Cornea.

[32] Bikbova G., Bikbov M. (2016). Standard corneal collagen crosslinking versus transepithelial iontophoresis-assisted corneal crosslinking, 24 months follow-up: randomized control trial. Acta Ophthalmol.

[33] Lombardo M., Giannini D., Lombardo G., Serrao S. (2017). Randomized controlled trial comparing transepithelial corneal cross-linking using iontophoresis with the Dresden protocol in progressive keratoconus. Ophthalmology.

[34] Sarma P., Kaur H., Hafezi F. (2023). Short-and long-term safety and efficacy of corneal collagen cross-linking in progressive keratoconus: a systematic review and meta-analysis of randomized controlled trials. Taiwan J Ophthalmol.

[35] Seyedian M.A., Aliakbari S., Miraftab M. (2015). Corneal collagen cross-linking in the treatment of progressive keratoconus: a randomized controlled contralateral eye study. Middle East Afr J Ophthalmol.

[36] Vandevenne M.M., Berendschot T.T., Winkens B. (2023). Efficacy of customized corneal crosslinking versus standard corneal crosslinking in patients with progressive keratoconus (C-CROSS study): study protocol for a randomized controlled trial. BMC Ophthalmol.

[37] Lang S.J., Messmer E.M., Geerling G. (2015). Prospective, randomized, double-blind trial to investigate the efficacy and safety of corneal cross-linking to halt the progression of keratoconus. BMC Ophthalmol.

[38] Nawaz S., Gupta S., Gogia V. (2015). Trans-epithelial versus conventional corneal collagen crosslinking: a randomized trial in keratoconus. Oman J Ophthalmol.

[39] Iqbal M., Gad A., Kotb A., Abdelhalim M. (2024). Analysis of the outcomes of three different cross-linking protocols for treatment of paediatric keratoconus: a multicentre randomized controlled trial. Acta Ophthalmol.

[40] Roszkowska A.M., Lombardo G., Mencucci R. (2023). A randomized clinical trial assessing theranostic-guided corneal cross-linking for treating keratoconus: the ARGO protocol. Int Ophthalmol.

[41] Greenstein S.A., Shah V.P., Fry K.L., Hersh P.S. (2011). Corneal thickness changes after corneal collagen crosslinking for keratoconus and corneal ectasia: one-year results. J Cataract Refract Surg.

[42] Price M.O., Fairchild K., Feng M.T., Price F.W. (2018). Prospective randomized trial of corneal cross-linking riboflavin dosing frequencies for treatment of keratoconus and corneal ectasia. Ophthalmology.

[43] Gomes J.A., Tan D., Rapuano C.J. (2015). Global consensus on keratoconus and ectatic diseases. Cornea.

[44] Kirillov A., Mintun E., Ravi N., Mao H., Rolland C., Gustafson L., Xiao T., Whitehead S., Berg A.C., Lo W.Y., Dollár P., Kirillov A., Mintun E., Ravi N. (2023).

[45] Fernández A., Garcia S., Herrera F., Chawla N.V. (2018). SMOTE for learning from imbalanced data: progress and challenges, marking the 15-year anniversary. J Artif Intelligence Res.

[46] He K, Zhang X, Ren S, Sun J (2016). Proceedings of the IEEE conference on computer vision and pattern recognition.

[47] Marcílio W.E., Eler D.M. (2020). From explanations to feature selection: assessing SHAP values as feature selection mechanism. In 2020 33rd SIBGRAPI conference on Graphics, Patterns and Images (SIBGRAPI).

[48] Selvaraju RR, Cogswell M, Das A, Vedantam R, Parikh D, Batra D (2017). Proceedings of the IEEE international conference on computer vision.

[49] Tellouck J., Touboul D., Santhiago M.R. (2016). Evolution profiles of different corneal parameters in progressive keratoconus. Cornea.

[50] Randleman J.B., Susanna B.N., Hammoud B., Dutra B.A., Scarcelli G., Santhiago M.R., Dupps W.J., Koch D.D. (2025). Evaluating the global consensus on keratoconus and ectatic diseases agreements reached on subclinical keratoconus. Am J Ophthalmol.

[51] Franco J., White C.A., Kruh J.N. (2020). Analysis of compensatory corneal epithelial thickness changes in keratoconus using corneal tomography. Cornea.

[52] Youssef A., Pencina M., Thakur A. (2023). External validation of AI models in health should be replaced with recurring local validation. Nat Med.

[53] Moons K.G., Kengne A.P., Grobbee D.E. (2012). Risk prediction models: II. External validation, model updating, and impact assessment. Heart.

[54] Wells B.J., Nguyen H.M., McWilliams A. (2025). A practical framework for appropriate implementation and review of artificial intelligence (FAIR-AI) in healthcare. NPJ Digital Med.

[55] Zhao X., Yuan Y., Sun T. (2022). Associations between keratoconus and the level of sex hormones: a cross-sectional study. Front Med.

[56] Gairola S., Bohra M., Shaheer N. (2021). Smartkc: Smartphone-based corneal topographer for keratoconus detection. Proc ACM Interact, Mobile, Wearable Ubiquitous Tech.

[57] Padmanabhan P., Elsheikh A. (2023). Keratoconus: a biomechanical perspective. Curr Eye Res.

